# PR Interval Associated Genes, Atrial Remodeling and Rhythm Outcome of Catheter Ablation of Atrial Fibrillation—A Gene-Based Analysis of GWAS Data

**DOI:** 10.3389/fgene.2017.00224

**Published:** 2017-12-19

**Authors:** Daniela Husser, Petra Büttner, Dorian Stübner, Laura Ueberham, Pyotr G. Platonov, Borislav Dinov, Arash Arya, Gerhard Hindricks, Andreas Bollmann

**Affiliations:** ^1^Department of Electrophysiology, Heart Center Leipzig, Leipzig University, Leipzig, Germany; ^2^Leipzig Heart Institute, Leipzig, Germany; ^3^Department of Cardiology, Clinical Sciences, Lund University, Lund, Sweden

**Keywords:** atrial fibrillation, PR interval, catheter ablation, genome wide association study, gene-based analysis

## Abstract

**Background:** PR interval prolongation has recently been shown to associate with advanced left atrial remodeling and atrial fibrillation (AF) recurrence after catheter ablation. While different genome-wide association studies (GWAS) have implicated 13 loci to associate with the PR interval as an AF endophenotype their subsequent associations with AF remodeling and response to catheter ablation are unknown. Here, we perform a gene-based analysis of GWAS data to test the hypothesis that PR interval candidate genes also associate with left atrial remodeling and arrhythmia recurrence following AF catheter ablation.

**Methods and Results:** Samples from 660 patients with paroxysmal (*n* = 370) or persistent AF (*n* = 290) undergoing AF catheter ablation were genotyped for ~1,000,000 SNPs. Gene-based association was investigated using VEGAS (versatile gene-based association study). Among the 13 candidate genes, *SLC8A1, MEIS1, ITGA9, SCN5A*, and *SOX5* associated with the PR interval. Of those, *ITGA9* and *SOX5* were significantly associated with left atrial low voltage areas and left atrial diameter and subsequently with AF recurrence after radiofrequency catheter ablation.

**Conclusion:** This study suggests contributions of *ITGA9* and *SOX5* to AF remodeling expressed as PR interval prolongation, low voltage areas and left atrial dilatation and subsequently to response to catheter ablation. Future and larger studies are necessary to replicate and apply these findings with the aim of designing AF pathophysiology-based multi-locus risk scores.

## Introduction

PR interval prolongation has recently been shown to associate with advanced left atrial remodeling expressed by left atrial (LA) dilatation and reduced LA voltage as well as atrial fibrillation (AF) recurrence after catheter ablation (Park et al., 2014). While different genome-wide association studies (GWAS) have implicated 13 loci to associate with the PR interval as an AF endophenotype (Holm et al., 2010; Pfeufer et al., 2010; Butler et al., 2012; Sano et al., 2014) their subsequent associations with electroanatomical remodeling and response to catheter ablation are unknown.

Genetic variants found in GWAS often extend over broad genomic distances, raising the possibility that those loci may contain multiple independent signals. Moreover, linkage disequilibrium (LD) structure and genotyping coverage may impact on GWAS results when only analyzing single SNPs. Consequently, gene-based association tests have been suggested for post-GWAS analysis and have been designed to detect genes that are genome-wide significant, but where no single SNP effect is large enough to be genome-wide significant by univariate tests.

We have previously applied gene-based testing to analyze the association between AF susceptibility genes *PITX2, KCNN3*, and *ZFHX3* and left atrial diameter (LAD), AF type and AF recurrence after ablation. Among the three candidate genes, only *ZFHX3* associated with LA dilatation and AF recurrence after catheter ablation (Husser et al., 2017).

Here, we perform a comparable gene-based analysis of GWAS data to test the hypothesis that PR interval candidate genes also associate with left atrial remodeling and arrhythmia recurrence following AF catheter ablation.

## Methods

### Patients

Six hundred-and-sixty patients with non-valvular AF undergoing de-novo radiofrequency AF catheter ablation between 2008 and 2013 were enrolled in the Leipzig Heart Center AF Ablation and Genetics Registry. The study protocol was approved by the Ethics Committee of the Leipzig University Medical Faculty and performed in accordance with the 1964 Helsinki declaration and its later amendments. Written informed consent was obtained from all individual participants included in the study.

Paroxysmal AF was defined as self-terminating episodes of AF within 7 days after onset documented by ECG or an ambulatory ECG monitor. Persistent AF was defined as an AF episode either lasting longer than 7 days or requiring drug or direct current cardioversion for termination.

Transthoracic and transesophageal echocardiography (TEE) was performed prior to catheter ablation. LAD and left ventricular ejection fraction were determined using standard measurements and a left atrial thrombus was excluded.

All class I or III antiarrhythmic medications with the exception of amiodarone were discontinued at least 5 half-lives before the procedure.

PR interval was determined from the digitally-acquired resting, supine, standard twelve-lead ECGs prior AF ablation and was defined as the duration from the earliest onset of the P wave to the earliest onset of the QRS complex (ms) in any of the12 standard ECG leads. Patients on amiodarone, with pacemaker stimulation, Wolff-Parkinson-White syndrome, or second/third degree heart block were excluded from this analysis.

### AF catheter ablation and follow-up

Left atrial catheter ablation was performed using a previously described approach (Husser et al., 2010; Dinov et al., 2014). In brief, patients were studied under deep propofol sedation with continuous invasive monitoring of arterial blood pressure and oxygen saturation. Non-fluoroscopic 3D catheter orientation, CT image integration, and tagging of the ablation sites were performed using Ensite NavX, Ensite Velocity (St. Jude Medical, St. Paul, MN, USA) or CARTO 3 (Biosense Webster, Diamond Bar, CA, USA). Trans-septal access and catheter navigation were performed with a steerable sheath (Agilis, St. Jude Medical, St. Paul, MN, USA). Patients presenting with AF at the beginning of the procedure were electrically cardioverted and ablation was performed during sinus rhythm (i.e., AF termination by ablation was not attempted). In all patients circumferential left atrial ablation lines were placed around the antrum of the ipsilateral pulmonary veins (irrigated tip catheter, pre-selected tip temperature of 48°C, and maximum power of 20–40 W). In patients with persistent AF, linear lesions were added at the left atrial roof, the basal posterior wall and the left atrial isthmus or in low voltage areas (LVA) until 2011. In patients recruited between 2011 and 2013, electro-anatomical voltage mapping to characterize LVA defined as potentials below 0.5 mV was performed as previously described (Dinov et al., 2014). After circumferential line placement, voltage and pace mapping along the ablation line were used to identify and close gaps. The isolation of all pulmonary veins with bidirectional block was verified with a multipolar circular mapping catheter and was defined as the procedural endpoint. Burst pacing from coronary sinus (down to 200 ms or atrial refractoriness) was performed at the end of the procedure. If sustained AF was induced, patients were electrically cardioverted and no additional ablation was performed. If atrial tachycardia was induced, those were mapped and ablated.

After ablation, class I and III antiarrhythmic drugs were not reinitiated. Oral anticoagulation was prescribed for 6 months, and proton pump inhibitors were added for 4 weeks. All patients were followed in the outpatient clinic for 12 months after the ablation. During this follow-up period, 7-day Holter ECG recordings were performed 3, 6, and 12 months after the ablation. Additional ECGs and Holter ECG recordings were obtained when patients' symptoms were suggestive of AF. AF recurrence was defined as a documented AF episode lasting longer than 30 s between 3 and 12 months after the ablation (thus, including a 3-month “blanking period”). All patients with sustained early recurring AF underwent electrical cardioversion. Additional drug administration was left to the discretion of the treating physician.

### Sample processing

Blood samples were obtained in EDTA test tubes in fasting state prior ablation. Genomic DNA was isolated using a commercial kit according to the manufacturer's recommendations (PeqLab, Erlangen, Germany). Genotyping was performed using HumanOmniExpressExome-8-v1.2 arrays comprising about one million Single Nucleotide Polymorphisms (SNPs) according to established protocols (Illumina, San Diego, US).

Genotyping call rate in all subjects was >95% except in three samples (<85%) that were excluded from further analysis.

### Data analysis and statistics

Raw data was compiled using GenomeStudio (Illumina) software and exported to PLINK GWAs analysis package version 1.9 (Purcell et al., 2007). Using PLINK tool set the data was tested for consistency. Samples with a call rate <95% were excluded. Single SNPs had to meet the following criteria: minor allele frequencies (MAF) >0.01, call rate >95%, Hardy-Weinberg equilibrium (HWE) significance threshold >0.0001. Otherwise they were excluded from further analysis.

Association of SNPs with PR interval and LAD was analyzed using linear regression and association of SNPs with LVA presence and arrhythmia recurrence was analyzed using logistic regression analysis. All analyses were adjusted for age, gender, and AF type.

Illumina's exome arrays contain specific “exm-SNPs” which were assigned to their corresponding dbSNP rs IDs prior further analysis.

We applied VEGAS (versatile gene-based association study) that uses the sum of *X* (Holm et al., 2010) for an individual SNP to generate a test statistic suggested for the gene. The *P*-value of the gene is then computed after accounting for LD and the number of SNPs in each gene but no effect measure (i.e., odds or hazard ratio) is provided (Liu et al., 2010). This is a more conservative test with a high specificity and very low proportion of false positives (Wojcik et al., 2015). SNPs within 5 kB of the candidate genes were selected for inclusion. This conservative flank was selected to exclude potential false positive signals from adjacent genes and “non-causal” SNPs which may impact on statistical power (Petersen et al., 2013).

We applied a three-stage analysis plan. First, we identified candidate genes that associated with PR interval. For this step, a Bonferroni correction was applied to account for the analysis of 13 genes (*P*-value less than 0.05/13 = 0.0038). Second, association of those identified gene(s) with markers of remodeling, i.e., LVA and LAD was analyzed. Finally, we tested associations with AF ablation outcome. Similarly, Bonferroni correction was applied to account for the number of genes and phenotypes.

Clinical variables are presented as mean ± one standard deviation or percentages. They were compared between patients with and without AF recurrence using chi-square or Student's *t*-test.

All relevant data are within the paper and its supporting documents. The datasets used and analyzed during the current study are available from the corresponding author on reasonable request.

## Results

### Patient characteristics

The study population included 660 patients (Table 1). Of those, PR interval was available in 209 patients and measured 180 ± 31 ms. Mapping information was available in 164 patients with LVA being present in 30%. AF recurrence between 3 and 12 months was observed in 48%. Patients with recurrence were older, had larger left atria, more often persistent AF and longer PR interval (Table 2).

**Table 1:** Patient characteristics.

**Cohorts**	**Total (*n* = 660)**	**PR interval (*n* = 209)**	**LVA (*n* = 164)**	***P*-value (ANOVA)**
Age (years)	60 ± 10	59 ± 10	61 ± 10	0.079
Male gender (%)	68	71	75	0.237
Body mass index (kg/m^2^)	29 ± 5	29 ± 4	29 ± 5	0.884
Idiopathic AF (%)	14	13	15	0.935
Persistent AF (%)	44	33	61	<0.001^a^
LAD (mm)	43 ± 6	42 ± 5	44 ± 6	0.006^b^
LVEF (%)	59 ± 10	60 ± 9	57 ± 10	0.006^b^

a *Significant difference between all cohorts*.

b *Significant difference between LVA vs. PR interval and total cohorts*.

**Table 2:** Patient characteristics in patients with and without AF recurrence^a^.

	**AF recurrence (*n* = 318)**	**No recurrence (*n* = 341)**	***P*-value**
Age (years)	61 ± 10	59 ± 10	0.007
Male gender (%)	70	67	ns
Body mass index (kg/m^2^)	29.2 ± 4.6	28.7 ± 4.5	ns
Persistent AF (%)	53	35	<0.001
LAD (mm)	44 ± 6	42 ± 6	<0.001
LVEF (%)	58 ± 10	59 ± 9	ns
PR interval (ms)^b^	186 ± 31	176 ± 30	0.027

a *One patient was lost to follow-up*.

b *Available in 209 patients*.

### Gene-based associations

**Figure 1:**
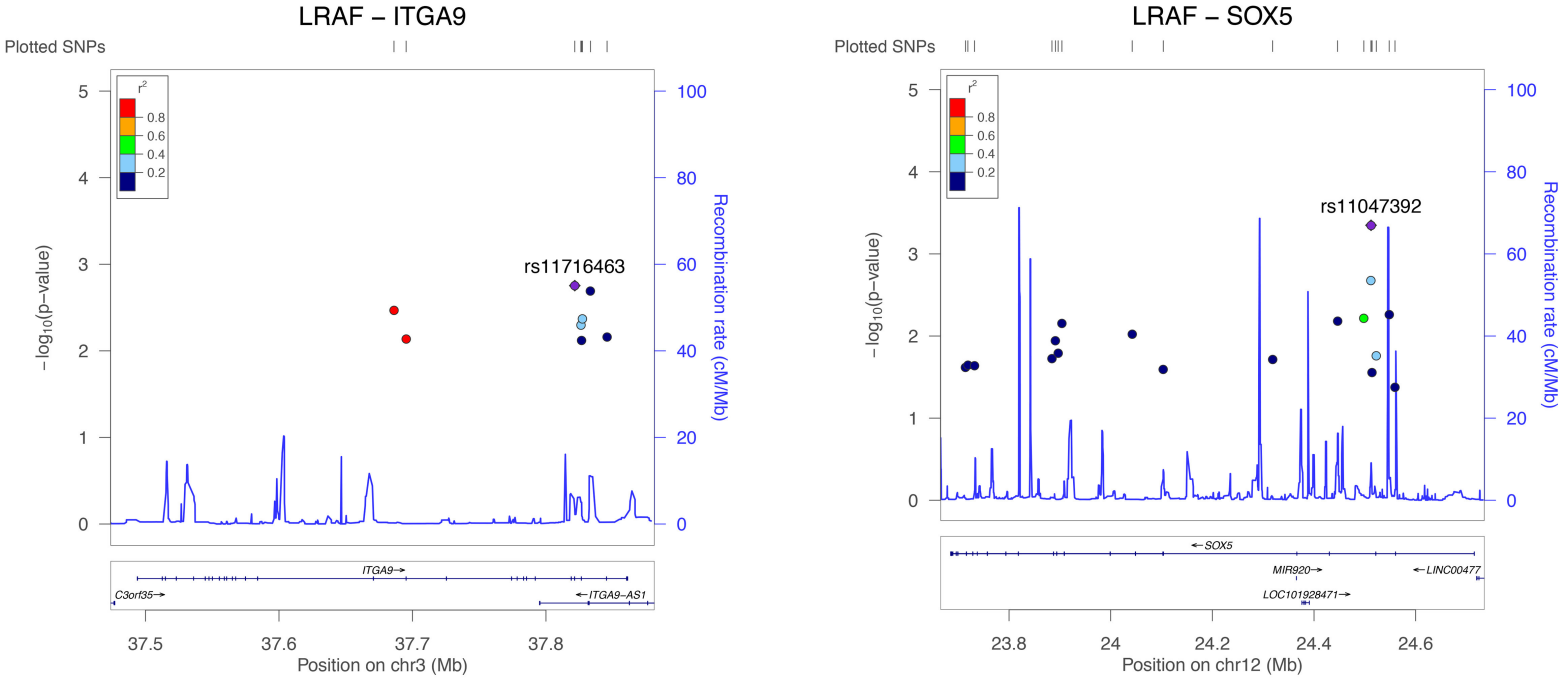
Regional association plot for all significant *ITGA9* (**left**) and *SOX5* (**right**) SNPs and response to AF ablation. The plot was created using GWAS association data that served as the input for gene-based testing. The *x*-axis represents the distribution of SNPs across the genes while the *y*-axis represents the −log_10_ of the *P*-value of each SNP in the gene. The colors indicate the *r*^2^ between the SNP with the lowest *P*-value and all the other SNPs. The light blue trace indicates recombination hotspots.

Results from the gene-based test for association between PR interval candidate genes and different phenotypes are summarized in Table 3. Among the 13 candidate genes, *SLC8A1, MEIS1, ITGA9, SCN5A*, and *SOX5* reached pre-defined significance threshold for association with the PR interval. In step two, those genes were then tested for their association with LVA und LAD. While *ITGA9* and *SOX5* were significantly associated with both remodeling phenotypes, *SLC8A1* was also associated with both phenotypes but did not reach the Bonferroni corrected *P*-value threshold ((*P*-value less than 0.05/5 genes ^*^ 2 phenotypes) = 0.005). Finally, both *ITGA9* and *SOX5* showed a significant association with AF recurrence (Table 3, Figure 1). Selection of different flank sizes did not impact on this association (Supplementary Table 1). Associated *ITGA9* (*n* = 8) and *SOX5* (*n* = 18) SNPs are listed in Supplementary Tables 2, 3.

**Table 3:** Gene-based association results.

**Gene**	**Chromosome**	**PR interval**	**LA LVA**	**LAD**	**AF recurrence**
*SLC8A1*	2	2,8E-03	1.7E-04	1.45E-02	3,96E-02
*MEIS1*	2	2,8E-04	ns	ns	ns
***ITGA9***	**3**	**1,0E-06**	**5,0E-06**	**2.51E-03**	**1,9E-05**
*SCN5A*	3	1,3E-04	1,0E-06	ns	ns
*SCN10A*	3	4,0E-02	2,3E-04	ns	ns
*ARHGAP24*	4	1,1E-02	ns	ns	5,0E-02
*NKX2-5*	5	ns	ns	3.96E-02	ns
*CAV2*	7	ns	ns	ns	ns
*CAV1*	7	ns	ns	9.57E-03	ns
*WNT11*	11	1,1E-02	ns	ns	ns
***SOX5***	**12**	**1,0E-06**	**1,0E-06**	**1,0E-06**	**1,0E-06**
*TBX3*	12	2,5E-02	ns	4.95E-02	ns
*TBX5*	12	ns	ns	2.13E-03	4,5E-04

*Genes in bold showed consistent association with different phenotypes*.

## Discussion

### Main findings

In this study, gene-based association of several atrial remodeling markers and AF ablation outcome was investigated in 660 patients in a three-stage analysis. Among 13 PR interval candidate genes, *SLC8A1, MEIS1, ITGA9, SCN5A*, and *SOX5* associated with the PR interval. Of those, *ITGA9* and *SOX5* were significantly associated with LA, LVA, and LAD and subsequently with AF recurrence after radiofrequency catheter ablation.

### Association between genotype, PR interval, atrial remodeling and AF ablation outcomes

Previous GWAS have identified several SNPs to associate with the PR interval (Holm et al., 2010; Pfeufer et al., 2010; Butler et al., 2012; Sano et al., 2014). Interestingly, the majority of the implicated candidate genes was also associated with the PR interval in our cohort (Table 3), although it reached pre-defined, Bonferroni-corrected significance threshold only for *SLC8A1, MEIS1, ITGA9, SCN5A*, and *SOX5*—probable due to sample size and use of conservative gene-based test.

Common genetic variants have also been associated with ablation outcome. Of those, the 4q25 variants have been shown the strongest association in single-center studies and a recent meta-analysis (Husser et al., 2010; Shoemaker et al., 2015) with radiofrequency and cryoablation (Miyazaki et al., 2017) although results have not been consistent (Choi et al., 2015). In this study, we focused on gene-based tests and in a step-wise approach that first identified an association between genotype and PR interval, than between genotype and LVA and LAD and finally between genotype and AF ablation outcome. This was based on the observations that (1) PR interval is in part determined by genotype (Holm et al., 2010; Pfeufer et al., 2010; Butler et al., 2012; Sano et al., 2014) and (2) prolonged PR interval is a marker of electroanatomical remodeling and ablation outcome (Park et al., 2014). Our study revealed associations of *ITGA9* and *SOX5* with (1) PR interval, (2) LAD, and LVA and (3) subsequently with rhythm outcome after ablation.

This study extends the concept of analyzing clinically-overlapping phenotypes with shared genotypes. By applying this, we were already able to identify genes of calcium signaling pathway (*SLC8A1*) and the ECM receptor interaction pathway (*ITGA9*) to associate with recurring AF (Husser et al., 2016a).

### *ITGA9* and *SOX5* as candidate genes for remodeling and response to AF ablation

*ITGA9* has been associated with the PR interval in African Americans (Butler et al., 2012). It has been speculated that *ITGA9* marks a distal *SCN5A* regulatory element (Butler et al., 2012) thereby affecting cardiac conduction although direct evidence is lacking. Although the 3p22–24 region has been linked with advanced conduction disease in Brugada syndrome (Weiss et al., 2002) further sequencing identified a single genetic variant in the *GPD1-L* gene that in turn reduced the sodium current via reduced *SCN5A* cell surface expression (London et al., 2007).

SNPs near *SOX5* have also previously been associated with PR interval in GWAS (Pfeufer et al., 2010) that is supported by the observation that response to rate control was also associated with *SOX5* (Kolek et al., 2014) although genome-wide significance was not reached. However, the mechanism by which this gene influences cardiac electrophysiology remains unknown.

Finally, *SLC8A1* was also associated with all analyzed phenotypes in our study but failed to reach Bonferroni-corrected significance threshold. This locus has recently been found to associate with PR interval in Asians (Sano et al., 2014) *SLC8A1* encodes Na(+)/Ca(2+) exchangers that contribute to Ca(2+)-homeostasis (Khananshvili, 2013). In *SLC8A1* knockout mice, their regulatory role of conduction of sinoatrial node, atrium and atrioventricular node has been demonstrated (Sano et al., 2014). In addition, cardiac-specific knockout of the Na(+)/Ca(2+) exchanger, NCX1, was associated with ventricular enlargement and reduced function (Henderson et al., 2004). Moreover, in humans, Ca(2+)-homeostasis has been identified as central contributor to different phenotypes of atrial electroanatomical remodeling and AF progression with a shared genomic background (Husser et al., 2016b).

Taken together, those and our findings implicate *ITGA9, SOX5* and potentially *SLC8A1* in several AF-remodeling associated processes that may underlie AF-remodeling and subsequently response to AF catheter ablation.

## Limitations

There are several limitations that are comparable to our previous study (Husser et al., 2017). This study included small sample size. However, it was based on PR interval candidate genes that were selected for gene-based analysis of GWAS data a priori. In other words, although we used GWAS data, we applied a hypothesis-driven approach. Furthermore, we addressed this by using well-defined intermediate AF phenotypes that are known interrelated markers of remodeling and subsequent AF ablation outcome and used a three-step analysis approach.

Ablation strategies in persistent AF are evolving and linear lesion sets have lessened in popularity due to no incremental benefit and even pro-arrhythmia. This may impact on type of arrhythmia recurrence and should be considered when assessing recurrence rates and comparing this to other studies.

This study does not contribute to the elaboration of molecular mechanisms which was beyond the scope of this study and did not address the question whether or not genotype impacts on proarrhythmic ablation effects or AF progression.

Finally, ablation outcome was assessed with serial clinical and prolonged Holter ECG monitoring which is in line with current recommendations but can nevertheless miss common asymptomatic AF episodes (Verma et al., 2013).

## Conclusions

This study suggests contributions of *ITGA9* and *SOX5* to AF remodeling expressed as PR interval prolongation, low voltage areas and left atrial dilatation and subsequently to response to catheter ablation. Future and larger studies are necessary to replicate and apply these findings with the aim of designing AF pathophysiology-based multi-locus risk scores.

## Author contributions

DH and AB did the conception of the study and wrote the manuscript. PB did the GWAS analysis. DS, LU, BD, and AA acquired the data. DS and PP performed ECG analysis, PP, PB, and GH participated in data interpretation and critically revised the manuscript. All authors read and assigned the manuscript and are accountable for the content.

### Conflict of interest statement

The authors declare that the research was conducted in the absence of any commercial or financial relationships that could be construed as a potential conflict of interest.
