# Supplementary material for: PR Interval Associated Genes, Atrial Remodeling and Rhythm Outcome of Catheter Ablation of Atrial Fibrillation—A Gene-Based Analysis of GWAS Data
Source: Front Genet. 2017 Dec 19;8:224. doi: 10.3389/fgene.2017.00224 (PMC5742186; doi:10.3389/fgene.2017.00224)
Supplement: Supplementary file 1 [file Table1.PDF]

**Supplementary Table 1.** Impact of different flank sizes on the association between *ITGA9* and *SOX5* with several AF phenotypes.

| | SNPs | | PR interval | | LA LVA | | LAD | | AF recurrence | |
|---|---|---|---|---|---|---|---|---|---|---|
| flank in kb | *ITGA9* | *SOX5* | *ITGA9* | *SOX5* | *ITGA9* | *SOX5* | *ITGA9* | *SOX5* | *ITGA9* | *SOX5* |
| 0 | 16 | 22 | 7,00E-06 | 1,00E-06 | 5,00E-06 | 1,00E-06 | 2,51E-03 | 1,00E-06 | 1,85E-05 | 1,00E-06 |
| 5 | 17 | 22 | 1,00E-06 | 1,00E-06 | 5,00E-06 | 1,00E-06 | 2,51E-03 | 1,00E-06 | 1,85E-05 | 1,00E-06 |
| 20 | 17 | 23 | 1,00E-06 | 1,00E-06 | 5,00E-06 | 1,00E-06 | 2,51E-03 | 1,00E-06 | 1,85E-05 | 1,00E-06 |
| 100 | 17 | 24 | 1,00E-06 | 1,00E-06 | 5,00E-06 | 1,00E-06 | 2,51E-03 | 1,00E-06 | 1,85E-05 | 1,00E-06 |
