# Supplementary material for: PR Interval Associated Genes, Atrial Remodeling and Rhythm Outcome of Catheter Ablation of Atrial Fibrillation—A Gene-Based Analysis of GWAS Data
Source: Front Genet. 2017 Dec 19;8:224. doi: 10.3389/fgene.2017.00224 (PMC5742186; doi:10.3389/fgene.2017.00224)
Supplement: Supplementary file 2 [file Tables2and3.pdf]

Supplementary Table 2. *ITGA9* SNPs associated with LRAF.

SNP.id	Risk allele	OR	95%	CI	P value
rs11129771	C	1.499	1.159	1.937	0.002029
rs11714019	G	1.997	1.257	3.172	0.00339
rs11716463	C	2.066	1.311	3.255	0.001765
rs17275376	G	1.689	1.155	2.47	0.00689
rs2685109	T	1.533	1.144	2.055	0.00426
rs2685111	C	1.502	1.13	1.995	0.005009
rs2844397	A	1.454	1.105	1.914	0.007581
rs57547208	G	1.893	1.188	3.016	0.007299

Supplementary Table 3. *SOX5* SNPs associated with LRAF.

SNP.id	Risk allele	OR	95%	CI	P value
rs1031895	G	1.307	1.038	1.647	0.02285
rs10505893	C	1.304	1.035	1.642	0.02411
rs10505894	T	1.31	1.039	1.653	0.02255
rs10505904	A	1.402	1.058	1.857	0.0187
rs10505912	C	0.5164	0.3135	0.8506	0.009444
rs10505917	C	0.7644	0.604	0.9673	0.02531
rs10743498	T	1.289	1.009	1.646	0.04189
rs10842220	A	0.7528	0.5972	0.9488	0.01616
rs11047382	T	0.5828	0.3963	0.857	0.006059
rs11047392	T	0.4721	0.3104	0.7178	0.0004481
rs11047402	C	0.4949	0.2772	0.8837	0.0174
rs4540919	C	0.7271	0.5768	0.9167	0.007018
rs482146	C	1.819	1.182	2.8	0.006567
rs521144	C	1.299	1.029	1.64	0.02764
rs550338	A	0.6525	0.497	0.8566	0.002106
rs7307101	G	0.7317	0.587	0.9121	0.005458
rs7966642	G	0.7422	0.5892	0.935	0.0114
rs7969863	T	1.425	1.059	1.916	0.01919
